# *In vitro* evaluation of modified surface microhardness measurement, focus variation 3D microscopy and contact stylus profilometry to assess enamel surface loss after erosive–abrasive challenges

**DOI:** 10.1371/journal.pone.0175027

**Published:** 2017-04-05

**Authors:** Milán Gyurkovics, Tommy Baumann, Thiago Saads Carvalho, Cristiane Meira Assunção, Adrian Lussi

**Affiliations:** 1 Department of Preventive, Restorative and Pediatric Dentistry, University of Bern, Bern, Switzerland; 2 Faculty of Dentistry, Department of Conservative Dentistry, Semmelweis University, Budapest, Hungary; 3 Federal University of Rio Grande do Sul, School of Dentistry, Pediatric Dentistry Division, Porto Alegre, Brazil; Tokyo Ika Shika Daigaku, JAPAN

## Abstract

The aim of the study was to compare surface loss values after erosion—abrasion cycles obtained with modified surface microhardness measurement (mSMH), focus variation 3D microscopy (FVM) and contact stylus profilometry (CSP). We cut human molars into buccal and lingual halves, embedded them in resin and ground 200 μm of enamel away. The resulting surfaces were polished. To maintain a reference area, we applied Block-Out resin to partly cover the enamel surface. The samples were incubated in artificial saliva (37°C; 1 h), then rinsed in deionized water (10 s) and dried with oil-free air (5 s). We immersed the specimens individually in 30 mL citric acid (1%, pH 3.6) for 2 min (25°C, 70 rpm dynamic conditions) before brushing them (50 strokes, 200 g) in an automatic brushing machine with toothpaste-slurry. We calculated the surface loss as per mSMH, by re-measuring the length of the same six indentations made before the abrasive challenge. The experiment consisted of five experimental groups that received between 2 and 10 erosion—abrasion cycles. Each group contained 15 specimens and samples in groups 1, 2, 3, 4 and 5 underwent a total of 2, 4, 6, 8 and 10 cycles, respectively. The resin was removed from the reference area in one piece under 10× magnification and the FVM and CSP were performed. Agreement between the methods was calculated with the intraclass correlation coefficient (ICC) and depicted in Bland-Altman plots. All methods presented a linear pattern of surface loss measurements throughout the experiment, leading overall to a strong, statistically significant correlation between the methods (ICC = 0.85; p<0.001). So, despite the different surface loss values, all methods presented consistent results for surface loss measurement.

## Introduction

Erosive tooth wear is an irreversible loss of dental hard tissues, which results from the interaction of dietary or endogenous acids and intra-oral abrasive forces [1–6]. It is common in developed societies [7–11], and early diagnosis and prevention are important [12, 13].

Various in vitro methods are used to assess the hard tissue surface loss caused by erosive tooth wear at an initial stage, where it ranges from nanometers to a maximum of a few micrometers. This initial stage includes the enamel surface loss, so if we would like to set up an early diagnosis of erosive tooth wear, it is highly important to be able to detect even a minor enamel loss.

In vitro methods capable to measure this minor enamel loss include, among others, the modified surface microhardness measurement (mSMH), focus variation three-dimensional microscopy (FVM) and contact stylus profilometry (CSP) [14]. Although there are several methods available, few comparative studies on these methods have been reported [2, 14–21]. Furthermore, some authors suggest that caution is needed when comparing the absolute values of different methods to measure the same surface [17, 21]. Different measuring methods, different settings or even different devices with the same method might result in different absolute surface loss values [17, 22]. To overcome this issue, it would be advisable to use relative values, which might render the results of different methods more comparable.

Nevertheless, the combined application of the different measurements is highly recommended as a means to overcome the limitations of the individual procedures [19, 22]. Thus, the main objective of the present study was to compare the enamel surface loss measured by the mSMH, FVM and CSP methods, using the same specimens. Our null hypothesis was that there is no difference between the absolute values of the various measurements.

## Materials and methods

### Preparation of enamel specimens

Caries-free human molars were selected from a pooled bio-bank of extracted teeth. All tooth extractions were carried out in dental practices in Switzerland (no water fluoridation, 250 ppm F− in table salt), and the teeth were stored in 1% (w/v) chloramine T trihydrate solution. Patients were duly informed about the possible use of their teeth for research purposes, and consent was obtained. Ethical approval was not necessary as the local ethics committee categorizes the pooled bio-banks as “irreversibly anonymized” samples. Approved guidelines and regulations of the local ethics committee (Kantonale Ethikkommission: KEK) were strictly adhered to.

A diamond saw (Isomet, 11–1180 Low Speed Saw, Buehler, Lake Bluff, IL, USA), was used to separate the crowns from the roots and the crowns were then cut into buccal and lingual halves with the same saw. These halves were embedded in resin (Paladur, Heraeus Kulzer GmbH, Hanau, Germany) in two cylindrical planar parallel molds [23, 24]. We removed the thinner mold (200 μm thick), and polished the thicker mold (7 mm thick) using a polishing machine (LaboPol-21, Struers, Ballerup, Denmark) with a series of silicon carbide paper disks of 18 μm, 8 μm, and 5 μm grain size, for 30 s each, under constant tap water cooling.

After each polishing step, we rinsed the blocks under running tap water and sonicated them for 3 min. In this way we obtained 75 specimens with flat ground enamel where the superficial 200 μm had been removed [8, 25]. All specimens were stored in a mineral solution at 4°C (1.5 mM CaCl_2_, 1.0 mM KH_2_PO_4_, 50 mM NaCl, pH 7.0) until they were used in the experiments [26]. At the start of the experiments, the specimens were polished again with a 5 μm grit silicon carbide disk for 5 s under constant cooling (LaboPol-6, DP-Mol Polishing, DP-Stick HQ, Struers, Ballerup, Denmark) to remove any possible precipitation from the mineral storage solution. Furthermore, all specimens had half of their surfaces covered with a flowable light-curing resin (LC BlockOut^®^, Ultradent Products Inc., South Jordan, UT, USA) to create a reference area. No prior etching was done and no adhesive was used.

### Erosion–abrasion cycles

This experiment consisted of five experimental groups each of which contained 15 specimens treated with between 2 and 10 erosion—abrasion cycles. Thus, samples in groups 1, 2, 3, 4 and 5 underwent a total of 2, 4, 6, 8 and 10 cycles, respectively. One erosion—abrasion cycle comprised incubation of the specimens in artificial saliva for 1 h followed by one erosive challenge and one abrasive challenge, as described below.

The specimens were individually incubated in artificial saliva [25] at 37°C, for 1 h and then rinsed in deionized water for 10 s before being dried with oil-free compressed air (5 s).

All specimens were individually submitted to an erosive challenge with 30 mL of 1% citric acid (pH 3.6) at 25°C for 2 min, under dynamic mixing conditions (70 rpm; Salvis AG, Reussbühl, Switzerland). The specimens were then rinsed with deionized water (20 s) and dried with oil-free compressed air (5 s). For the abrasive challenge the samples were then brushed in an automatic brushing machine with 50 strokes (Syndicad Ingenieurbüro, München, Germany) using an American Dental Association (ADA) approved medium toothbrush with a 200 g load and toothpaste slurry that consisted of 1:1 (w/w) deionized water and fluoridated dentifrice (1350 ppm F; Radioactive Dentin Abrasion /RDA/ ca. 70; M-Budget Toothpaste, Migros, Zürich, Switzerland). The samples were kept for 2 min in the slurry, during which time the brushing procedure (25 s) was performed. After brushing, the specimens were carefully rinsed with deionized water and dried for 5 s with oil-free air. Thus, specimens underwent a total of 4, 8, 12, 16 or 20 min erosion; and 100, 200, 300, 400 or 500 toothbrush strokes in groups 1, 2, 3, 4 and 5, respectively. All samples were stored in a humid chamber between the cycles.

### Enamel surface loss calculated using the mSMH

Enamel surface loss was first calculated using the mSMH, which consists of measuring indentation lengths prior to and after abrasion [11, 27]. For this, we made six Knoop indentations on the exposed enamel surface using a Knoop microhardness tester immediately before an abrasive challenge (UHL VMHT Microhardness Tester, UHL technische Mikroskopie GmbH & Co. KG, Aßlar, Germany). Indentations were made at intervals of 25 μm, using a load of 50 g and dwell time of 10 s. Using the integrated software of the microhardness tester, we measured the length (L_1_) of these indentations and calculated their depths using the following equation: D_1_ = 0.5 × L_1_ × tan α, where α = 3.75°, a constant parameter of the Knoop diamond indenter. Immediately after the abrasive challenge, we re-measured the lengths (L_2_) of the same six indentations and calculated their new depths (D_2_). This procedure was repeated at every abrasive challenge, for every experimental cycle.

After each experimental cycle, enamel surface loss (ΔD_i_) was calculated using the formula ΔD_i_ = D_1_ − D_2_, where *i* is the *i*th experimental cycle, and D_1_ and D_2_ are the depths before and after the *i*th abrasive challenge.

This method has an accuracy of 0.18 μm, which is the standard deviation (SD) value of the average surface loss during one erosion—abrasion cycle calculated from 10 indentations. Furthermore, the precision was 0.01 μm and 0.08 μm, calculated from the SD values of the average depths of the same indentation measured 10 times before and 10 times after the abrasion, respectively (unpublished results).

### Enamel surface loss calculated by FVM

**Fig 1:**
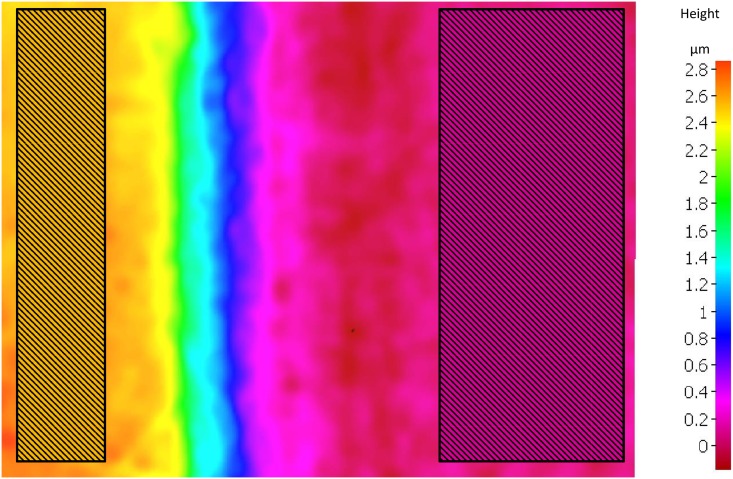
Illustration of the FVM measurement. The figure represents a typical output of the FVM (constant field of view 110 μm x 144 μm). The different colors represent the different heights on the surface of the specimen (see scale on the right). For measurement, rectangular areas are selected on both the reference surface (yellow) and the eroded-abraded surface (magenta). The software calculates the average height of these two selected areas, from these we calculate the amount of enamel lost.

After all experimental cycles, we removed the resin covering the reference area. A visual check was made under 10× magnification. Surface loss was then measured with FVM, which is a kind of optical profilometry based on the focus variation concept, to calculate the surface loss. Topographical images of the enamel surfaces were captured in three dimensions at a magnification of 200× using the InfiniteFocus^®^ G4 Microscope (IFM, Alicona Imaging, Grambach, Graz, Austria). The profile of the measurement surface was captured using computer software. In short, optical microscopic images are captured continuously as the distance between the object and objective is varied [28]. Owing to the small depth of field of the optics and the 3D structure of the specimen, only certain regions in each picture will appear as sharp images. Algorithms convert this variation of focus into 3D information, from which information on depth can be extracted [14, 27, 29]. In our study the following settings were used. The objective was IFM G4 100x (realized magnification: 98.7695). The instrument had a field of view of 144 μm × 110 μm and coaxial illumination was applied. The exposure time was optimized for each measurement and was between 0.8 and 1.5 ms, and the contrast values between 0.9 and 1.1. The lateral and vertical resolution of the device were 1.5 μm and 10 nm, respectively. During the measuring process, a rectangular area is selected on both the reference surface and the abraded surface: from these, the software calculates the average height differences of the selected areas. Fig 1 shows a typical example how the areas were selected manually by the samples. Variations in the X and Y values of the selected areas were ca. 20% and 5%, respectively (Fig 1). This average height difference was considered to represent enamel surface loss (expressed in μm) [21, 30].

This method has an accuracy of 0.05 μm, which is the SD value of the average surface loss during one erosion—abrasion cycle calculated from 6 independent fields of view on the same sample. Furthermore, the precision was 0.006 μm, which is the SD value of the average surface loss calculated from an analysis of the same field of view with 6 repeated adjustments of the rectangular areas (unpublished results).

### Enamel surface loss calculated by CSP

Enamel surface loss was also measured using a contact stylus profilometer (Perthometer S2, Mahr GmbH, Göttingen, Germany). The stylus had a diamond tip with a radius of 2.00 μm and the testing force was 0.7 mN. We performed six tracings, each of 3 mm in length, from the reference area to the abraded area, at intervals of 25 μm (stylus speed of 0.1 mm/s).

**Fig 2:**
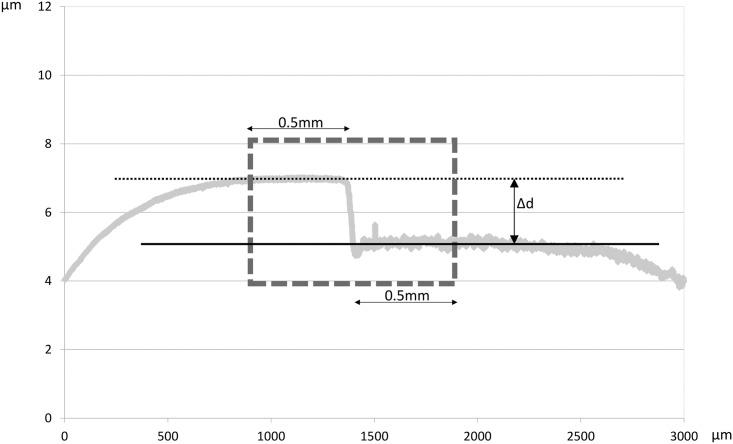
Illustration of the CSP profile. We selected a 1 mm segment around the step (comprising 0.5 mm of the reference area and 0.5 mm of the abraded area; dashed square line). Within this segment, one regression line was fitted to the reference area (dotted line) and one to the abraded area (continuous line). The average vertical distance (Δd) between the two regression lines was calculated for the selected range.

For each profile tracing, we constructed one regression line on the reference area and one on the abraded area. We then selected a segment of 1 mm around the step (comprising 0.5 mm of the reference area and 0.5 mm of the abraded area), and calculated the average vertical distance between the two regression lines within this 1 mm segment. The mean vertical distance from the six profiles of one specimen was considered as enamel surface loss, which was calculated in μm (Fig 2).

This method has an accuracy of 0.03 μm, which is the SD value of the average surface loss during one erosion—abrasion cycle calculated from 10 tracings. Furthermore, the precision was 0.01 μm, which is the SD value of the average surface loss calculated from an analysis of the same tracing repeated 10 times (unpublished results).

### Statistical analysis

Statistical analysis was performed with IBM SPSS Statistics v. 22 (IBM, Armonk, NY, USA). Descriptive statistics were used to define the median and the interquartile range of the surface loss values for each group, obtained using the different measurement techniques. Measurements with FVM and CSP were not possible if the reference and experimental area were separated by the embedding resin or the combined length was less than 3 mm. Differences between the measurement techniques (within the same group) were analyzed with the Friedman Test followed by a post-hoc Wilcoxon signed ranks test. Differences between groups (obtained using the same measurement technique) were analyzed using Mann-Whitney tests. For multiple comparisons we used Bonferroni corrections.

**Fig 3:**
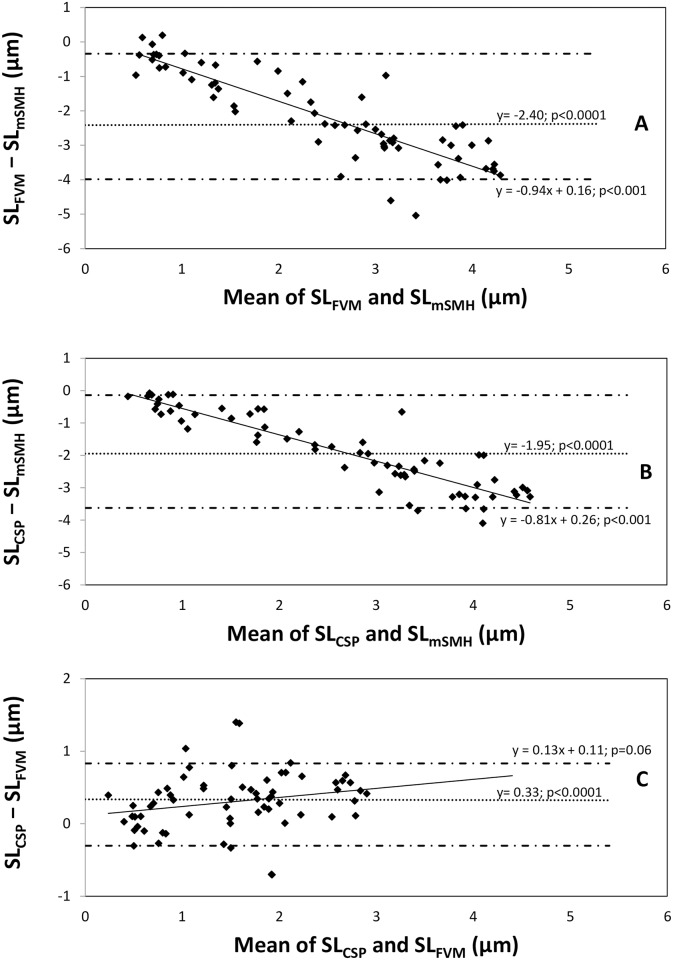
Bland-Altman plots assessing agreements between the different methods. “A” is the plot comparing FVM and mSMH, “B” stands for CSP versus mSMH and “C” compares CSP and FVM. Solid lines represent ordinary least square regression models (y = mx + c), where the slope (m) represents proportional bias when p < 0.05. Dotted lines represent the systematic differences, where relative bias is assumed when p < 0.05. The dotted and dashed lines represent 95% confidence intervals of the systematic difference (dotted) line. SL: surface loss.

Associations between mSMH, FVM and CSP were calculated with intra-class correlation coefficients (ICC) and depicted in Bland-Altman plots with nonparametric approach [31]. For ICC, a value near 0 indicates a low level of agreement, while a value close to 1 indicates a high level of agreement between the methods. In analyzing the Bland-Altman plots [22, 31, 32], proportional bias was assumed when the slopes (m) of the displayed regression lines (solid lines in Fig 3) were significantly different from 0. Relative bias was assumed when the mean value of the differences between two methods (dashed line in Fig 3) was significantly different from 0, according to a one-sample Wilcoxon Signed Rank Test [22, 32].

For all analyses, the significance level was set at 0.05.

## Results

**Fig 4:**
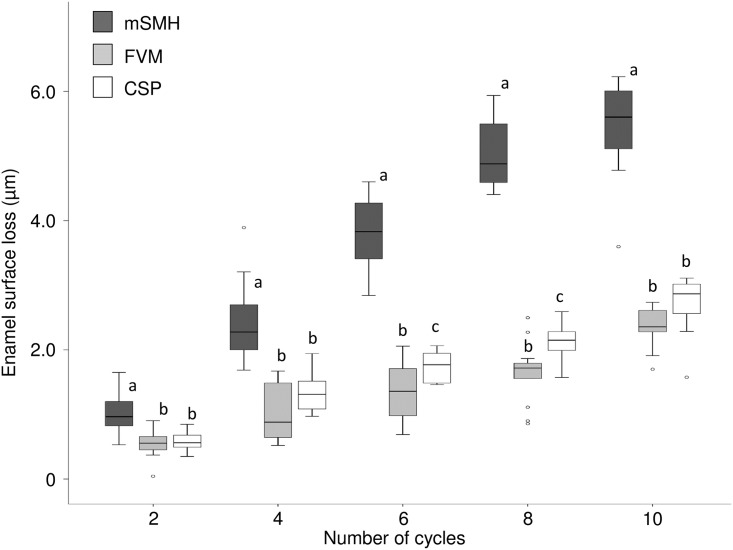
Box plots representing enamel surface loss values as a function of cycle numbers. Enamel surface loss median and interquartile values in μm according to the different cycle numbers (groups) using the three different methods. Different lower-case letters (a, b, c) represent significant differences between different methods after the same number of cycles (p<0.05).

In the present erosion—abrasion study, all methods were able to measure the enamel surface loss, although there were differences in the measured absolute values. Fig 4 shows the median and interquartile values of surface loss after the different erosive—abrasive cycles (groups) measured with each of the methods. All groups presented significantly greater surface loss values with consecutive cycles (p<0.05). Only for mSMH, there was no significant differences between surface loss after 8 and 10 cycles, and for FVM, there was no significant difference in surface loss between 6 and 8 cycles. Considering each group individually, there were significant differences in the surface loss values between mSMH and FVM (p<0.01) and between mSMH and CSP (p<0.05) for all groups. Comparison between FVM and CSP showed a significant difference after 6 (p<0.05) and 8 cycles (p<0.05).

Despite the differences described above, there was a strong general correlation of surface loss values between all methods (ICC = 0.85; p<0.0001). Analyzing the different methods in pairs, we observed moderate to strong correlations between FVM and mSMH: ICC = 0.7, CSP and mSMH: ICC = 0.8, and FVM and CSP: ICC = 0.93; p<0.0001. Using the Bland-Altman plots, which assess agreement between the different methods, mSMH presented significant proportional and relative bias compared to the other methods (Fig 3A and 3B). According to the significant proportional bias, the difference between the FVM and mSMH and between the CSP and mSMH methods increases as the average surface loss values increase. Fig 3C shows that there was no proportional bias between FVM and CSP (p = 0.06), but there was a significant relative bias (p<0.001).

## Discussion

We compared the enamel surface loss values measured by the mSMH, FVM and CSP methods, for different numbers of successive erosion—abrasion cycles (S1 File). CSP and mSMH are contact methods, while FVM is an optical surface analysis (non-contact) method. The latter has the advantage of being non-damaging and sometimes scans take only a few seconds [28, 29]. FVM is able to make a three-dimensional topographic surface analysis directly by optical imaging, with a vertical resolution of up to 10 nm [14, 29, 30]. This technique is ideal for the measurement of surface roughness and maximum peak-to-valley distance or for measuring the effect of toothpastes on enamel [30, 33–35]. Moreover, steep vertical structures can also be captured with FVM [14, 28].

With respect to CSP, concerns have been expressed that the diamond tip of the instrument may harm the surface of the specimen [12, 14, 17]. However, Heurich et al. showed that, even when the depths of these scratches were taken into consideration during surface loss studies, they had no significant influence on the depth values [17]. The profilometer used in our study applied a force of only 0.7 mN, and it had a stylus tip radius of 2 μm, both parameters being quite similar to those Heurich et al. used in their experiment (i.e. 0.87 mN and 2 μm) [17]. Therefore, the depths of the scratches on our specimens were most probably negligible and thus cannot explain the differences observed between mSMH and CSP.

The indentation method (mSMH) that we investigated is also robust and well-described [14, 22]. The average length of all the indentations was 45.53 ± 2.51 μm before and 27.51 ± 6.56 μm after abrasion. This resulted in an average surface loss of 0.59 ± 0.20 μm (≈0.6 μm) per cycle. This means that if we consider the average enamel thickness in case of molars 1.5 mm, then dentine would be reached in approximately 2500 cycles (2500x0.6 = 1500).

All the methods used in our investigation were therefore able to detect the enamel surface loss, although there were significant differences in the measurements obtained using the different methods. The Bland and Altman plots (Fig 3) show systematic differences in the measurements for each pair of methods: FVM and mSMH (Fig 3A), CSP and mSMH (Fig 3B), and CSP and FVM (Fig 3C). The solid lines represent ordinary least square regression models. If two given methods were to measure the same values, this line would run through 0, meaning that there is no difference between the methods being investigated (SL_FVM_ − SL_mSMH_ = 0, SL_CSP_ − SL_mSMH_ = 0, SL_CSP_−SL_FVM_ = 0; SL: surface loss). In our study, there was always a difference between the measured values of all the method pairs compared. Despite the observed differences between these average values, all comparison pairs showed a strong correlation according to the ICC values (0.7, 0.85 and 0.93 for FVM versus mSMH, CSP versus mSMH and CSP versus FVM, respectively). The Bland-Altman plot for CSP and FVM (Fig 3C) had a relatively small slope (m = 0.13) as compared with the slopes for FVM and mSMH (Fig 3A; m = −0.94) and CSP and mSMH (Fig 3B; m = −0.81). Furthermore, the proportional bias for CSP and FVM (Fig 3C; p = 0.06) was not significant. This means that as mean SL increased, we observed a greater difference between mSMH and the other two methods (Fig 3A and 3B; downward slope), whereas no significant differences were observed between CSP and FVM, as the SL progressed (Fig 3C; no significant slope). The correlation between CSP and FVM turned out to be the highest (ICC = 0.93). For both the FVM versus mSMH (Fig 3A) and CSP versus mSMH (Fig 3B) comparisons, the proportional bias was significant (p<0.0001).

An earlier experiment showed a positive correlation between CSP and FVM [21]. The authors also pointed out that the various types of methods might result in different surface loss values [21]. CSP was also compared to FVM by Ren et al., in their study assessing erosive surface loss of enamel. They concluded that although these two methods differ in the values measured [34], a statistically significant correlation could be observed [34]. Fujii et al. also found a good correlation between the two methods for enamel roughness measurements after erosion [28].

Some of the differences observed between the results obtained using contact and non-contact methods like CSP and FVM may arise because the results of optical measurements are influenced not only by purely geometrical changes in the surface but also by chemical changes induced by processing, which might influence the local refraction index of the surface [21].

Thus, different methods are bound to produce very different raw values for surface loss, but this does not mean that one technique is superior to another. All of them have their advantages and their disadvantages. They definitely depend on the surface features of the specimens, so flat and highly polished samples are necessary for accurate measurements [14, 17, 19, 21, 34]. CSP and mSMH show very robust and reproducible results, and both have been widely used in dental research [14]. Additionally, mSMH is a well-established technique that can be combined with abrasive surface loss measurements [14]. Moreover, CSP is also a well-established technique often applied to detect surface loss with high precision [14, 17]. By contrast, FVM is quite new and less well-studied, but it has the obvious advantage that it is a non-contact method, unlike the CSM and mSMH. FVM and CSP are more sensitive and they presented less variation for very initial surface loss after two cycles (see Fig 4), whereas mSMH had more variation in our study.

Notwithstanding these advantages and disadvantages, we emphasize that all the methods investigated in our study have been widely used in previous studies and have produced valid results for measurements of dental hard tissue loss. According to the relative differences, we observed that all three methods show very similar, linear results. In our study, taking the total amount of surface loss obtained in group 5 as 100%, we observed relative surface loss values ranging between 17% and 24% (for group 1), 37–46% (group 2), 57–69% (group 3), and 73–83% (group 4), which are presented in Table 1.

**Table 1:** Enamel surface loss median values in percentage according to the different groups as measured using the three different methods.

Group No.	No. of Cycle	Median in percentage (IQR)		
		mSMH	FVM	CSP
1	2	17 (15–21)	24 (19–28)	20 (17–24)
2	4	43 (36–46)	37 (28–63)	46 (38–53)
3	6	69 (63–78)	57 (48–70)	62 (53–67)
4	8	83 (82–98)	73 (65–73)	75 (70–79)
5	10	100 (91–108)	100 (97–111)	100 (89–105)

Relative surface loss values were calculated for each method in function of the total surface loss (group 5, 100%).

This is an important consideration when comparing different studies that used different methods to analyze surface loss. In such cases, relative values are more relevant than making direct comparisons between the absolute values.

As all methods presented linear results, we can extrapolate the data towards the very early stages of enamel erosive wear, and it is highly probable that we would be able to measure surface loss after one cycle. Therefore, if one is interested in the very early stages of erosive tooth wear, all the tested methods would be suitable for this measurement. In our setup, group 1, which underwent two cycles, represents the earliest stage investigated, in which we could clearly measure a significant surface loss with all methods already. Even earlier stages of erosive tooth wear might be investigated if a group undergoing only one cycle would be included. According to unreported data that we obtained by means of mSMH, the average surface loss after only one cycle was 0.59 μm. This supports the idea that only a single cycle could be performed to investigate very early stages on the one hand, and it perfectly correlates with the results of the later cycles on the other hand as well.

We therefore reject our original null hypothesis and conclude that there is a difference in the absolute values of surface loss when measured using the three investigated methods. Despite the different absolute surface loss values, all three techniques can provide consistent relative surface loss data.

## Supporting information

S1 File Raw data of the experiment. This file includes all the measurements received by the 75 samples. It contains the name of the sample, cycle number and enamel surface loss value in μm according to the applied technique (mSMH, FVM, CSP).
(XLSX)
